# Synergistic effects of 15-deoxy Δ^12,14^-prostaglandin J_2_ on the anti-tumor activity of doxorubicin in renal cell carcinoma

**DOI:** 10.1016/j.bbrep.2016.11.004

**Published:** 2016-11-17

**Authors:** Yasuhiro Yamamoto, Takehiro Yamamoto, Hiromi Koma, Ayaka Nishii, Tatsurou Yagami

**Affiliations:** a Division of Physiology, Department of Pharmaceutical Health Care, Faculty of Pharmaceutical Sciences, Himeji Dokkyo University, 2-1, kami-ohno 7-Chome, Himeji, Hyogo 670-8524, Japan; b Hyogo Prefectural Kobe High School, 1-5-1 Shironoshita-dori, Nada-ku, Kobe, Hyogo 657-0804, Japan

**Keywords:** Renal cell carcinoma, 15-deoxy Δ^12,14^-prostaglandin J_2_, Doxorubicin, Phosphoinositide 3-kinase, Chemoresistant

## Abstract

An endogenous anticancer agent, 15-deoxy -Δ^12,14^-prostaglandin J_2_ (15d-PGJ_2_) induces apoptosis in the chemoresistant renal cell carcinoma (RCC). Peroxisome proliferator-activated receptor-γ (PPARγ) is a nuclear receptor for 15d-PGJ_2_, and mediates the cytotoxicity of 15d-PGJ_2_ in many cancerous cells. However, 15d-PGJ_2_ induces apoptosis independently of PPARγ in human RCC cell line such as Caki-2. In the present study, we found that 15d-PGJ_2_ ameliorated the chemoresistance to one of anthracycline antibiotics, doxorubicin, in Caki-2 cells. Doxorubicin alone exhibited weak cytotoxicity at the concentrations effective for other cancer cells such as Hela cells. In addition, it did not activate caspase 3. However, the cytotoxicity of doxorubicin was increased remarkably and accompanied with the caspase- 3 activation in the presence of 15d-PGJ_2_. Doxorubicin alone damaged plasma membrane, and the combined application of 15d-PGJ_2_ with doxorubicin increased the membrane permeability slightly. PPARγ was involved in neither the anti-tumor activity nor the synergistic effect of 15d-PGJ_2_. 15d-PGJ_2_ induces apoptosis in Caki-2 cells via suppressing the phosphoinositide 3-kinase (PI3K)-Akt pathway. The effect of PI3K inhibitor on the cytotoxicity of doxorubicin was additive, but not synergistic. Although the PI3K inhibitor mimicked the cytotoxicity of 15d-PGJ_2_, it might not be involved in the synergism between 15d-PGJ_2_ and doxorubicin. In conclusion, 15d-PGJ_2_ enhanced the chemosensitivity of doxorubicin via the pathway independent of PPARγ and PI3K.

## Highlights

• 15d-PGJ_2_ increased cytotoxicity of doxorubicin markedly.
• PPARγ did not mediate the anti-tumor activity and the enhancing effect of 15d-PGJ_2_.
• 15d-PGJ_2_ and doxorubicin activated caspase 3 synergistically.
• The PI3K inhibitor mimicked the cytotoxicity of 15d-PGJ_2_.
• PI3K was not involved in the synergism between 15d-PGJ_2_ and doxorubicin.

## 1 Introduction

Renal cell carcinomas (RCCs) account for approximately 2% of adult carcinomas. Despite extensive evaluation of many different treatment modalities, advanced metastatic RCC remains highly resistant to radiotherapy and chemotherapy [1]. Clear cell RCC accounts for the majority of RCC cases [2] and one-third of the patients present with metastases at initial diagnosis. Nearly half of all patients with RCC die within 5 years of diagnosis and 5-year survival for those with metastatic disease is <10% [3]. Chemotherapeutic agents, such as gemcitabine, 5-fluorouracil (5-FU), capecitabine and vinblastine, exhibit clinical benefits partially [4]. Based on the immunogenicity of RCCs, the potency of cytokines, mainly interleukin 2 and/or interferon-α, have been reported by several clinical studies [5], [6]. The treatment of RCCs has been modified by chemotherapeutic agents, such as tyrosine kinase inhibitors (sunitinib, sorafenib, pazopanib, and axitinib), the anti-VEGF monoclonal antibody (bevacizumab) administered with interferon α) and mammalian target of rampamycin (mTOR) inhibitors (everolimus and temsirolimus) [7]. However, despite these novel therapies, the clinical outcome of patients with RCC remains poor [4].

To overcome the resistance of RCCs to chemotherapy, we have studied combinations of chemotherapy with new agents. Responsiveness of RCCs such as Caki-2 cell for conventional anticancer agents such as 5-FU, camptothecin (CPT) and etoposide (VP16) was lower than that of other types of cancer such as Hela cells [8], [9], [10], [11], [12]. CPT and VP16 are inhibitors of DNA topoisomerase I and II, respectively. DNA topoisomerases resolve topological constraints that may arise from DNA strand separation and are therefore important for transcription and replication [13]. Previously, we have reported that 15‑deoxy-Δ^12,14^‑prostaglandin J_2_ (15d-PGJ_2_) enhanced the anti-tumor activity of camptothecin, [11] and etoposide [12]. 15d-PGJ_2_ is an endogenous anticancer agent. Although peroxisome proliferator-activated receptor-γ (PPARγ) is a nuclear receptor for 15d-PGJ_2_[14], [15], it does not mediate the cytotoxicity of 15d-PGJ_2_ in RCCs [16], [17]. Furthermore, synergistic toxicities of 15d-PGJ_2_ with topoisomerases were also independent from PPARγ.

In cancer, the phosphoinositide 3-kinase (PI3K)/Akt and mTOR pathway is activated via multiple mechanisms [18]. Since the PI3K signaling is hyperactivated in RCCs, this pathway is one of targeted therapies [19]. 15d-PGJ_2_ inhibits proliferation of primary astrocytes [20] and neuroblastoma x DRG neuron hybrid cell line N18D3 [21] via down-regulating PI3K-Akt pathway. Previously, we have reported that the PI3K/Akt signaling mediated the cytotoxicity of 15d-PGJ_2_[17]. Here, we found that a PI3K inhibitor, LY294002, mimicked the cytotoxicity of 15d-PGJ_2_. Furthermore, 15d-PGJ_2_ enhanced the anti-tumor activity of the anthracycline antibiotic, doxorubicin, synergistically. In the present study, we ascertained whether the PI3K inhibitor enhanced the anti-tumor activity of doxorubicin.

## 2 Materials and methods

### 2.1 Cell lines and cell culture

The Caki-2 human RCC cell line was obtained from Summit Pharmaceuticals International (Tokyo, Japan). The Caki-2 cells were routinely cultured in RPMI-1640 medium supplemented with 10% fetal bovine serum, 50 mg/l penicillin G and 50 mg/l streptomycin (Invitrogen, Tokyo, Japan), at 37 °C in a 5% CO_2_-95% room air.

### 2.2 Reagents

15d-PGJ_2_ was obtained from Cayman Chemicals (Ann Arbor, MI; Cabru, Milan, Italy). Doxorubicin was purchased from Wako Pure Chemical Industries, Ltd. (Osaka, Japan). GW9662 was obtained from Sigma-Aldrich (St. Louis, MO, USA). 3-(4,5-dimethylthiazol-2-yl)−2,5-diphenyl tetrazolium bromide dye (MTT) and propidium iodide (PI) were purchased from Dojindo Laboratories (Kumamoto, Japan). LY294002 was purchased from Cell Signaling Technology (Boston, MA). The protein concentration was measured using the BCA protein assay reagent obtained from Pierce (Rockford, IL).

### 2.3 Cell viability analysis

15d-PGJ_2_ was dissolved in culture medium after evaporation of vehicle. Two different methods were employed for assessment of cell viability as previously reported. First, the MTT reduction assay reflecting mitochondrial succinate dehydrogenase activity was employed. The cells were seeded on a 96-well tissue culture plate at 10,000 cells/cm^2^ and incubated for 24 h prior to drug exposure. The cells were incubated with 15d-PGJ_2_ and doxorubicin at the indicated concentrations. After 20 h or 24 h, the cells were incubated with MTT solution (0.1 mg/ml in phosphate-buffered saline) for an additional 3 h at 37 °C. The MTT solution was then aspirated off. To dissolve the formazan crystals formed in viable cells, 100 μl dimethyl sulfoxide was added to each well. Absorbance was measured at 570 nm using a spectrophotometer (iMark Microplate Reader, Bio-Rad Laboratories, Hercules, CA, USA). Second, Cell death was also measured by manually counting the percentage of neurons that stained with propidium iodide (PI, 0.1 µg/ml). The cells were seeded on a 24-well tissue culture plate at 10,000 cells/cm^2^ and incubated for 24 h prior to drug exposure. Nuclei stained with PI were counted from 12 fields with data expressed as percentage PI-stained cells normalized to the vehicle-treated group.

### 2.4 Fluorimetric assay of caspase-3 activity

Caspase-3 activity was assessed using a Caspase-3 Fluorimetric Assay kit, (Sigma-Aldrich), according to the manufacturer's instructions. Briefly, the cells were seeded into 96-well plates at a density of 10,000 cells/cm^2^ and incubated with 20 μM 15d-PGJ_2_ and 1 μM doxorubicin for 24 h. After exposure to the drugs for 24 h, the supernatants were aspirated and the cells were harvested with lysis buffer [50 mM HEPES (pH 7.4), 5 mM CHAPS and 5 mM DTT]. The reaction buffer, including acetyl-Asp-Glu-Val-Asp-7-amido4-methylcoumarin (Ac-DEVD-AMC), a caspase-3 specific substrate, was added to the wells and the production of AMC was sequentially detected with a CytoFluor® Plate reader (MTX Lab Systems, Vienna, VA, USA) at an excitation wavelength of 360 nm and at an emission wavelength of 460 nm. The enzyme activities were determined as initial velocities expressed as nmol AMC/min/ml and were then corrected by the quantity of protein in each well detected by bicinchoninic acid protein assays (Thermo Fisher Scientific, Waltham, MA, USA).

### 2.5 Statistical analysis

Data are given as means±SE (n=numbers of observations). We performed two experiments at least on different days, and confirmed their reproducibility. We analyzed observations obtained on the same day, and presented the typical experimental results among independent ones on different days to minimize experimental errors. Data were statistically analyzed with the Student's *t*-test for comparison with the control group. Data on various drugs were statistically analyzed by two-way ANOVA followed by Dunnett's test for comparison between the groups.

## 3 Results

### 3.1 15d-PGJ_2_ exacerbated the doxorubicin-disrupted morphology in Caki-2 cells

**Fig. 1:**
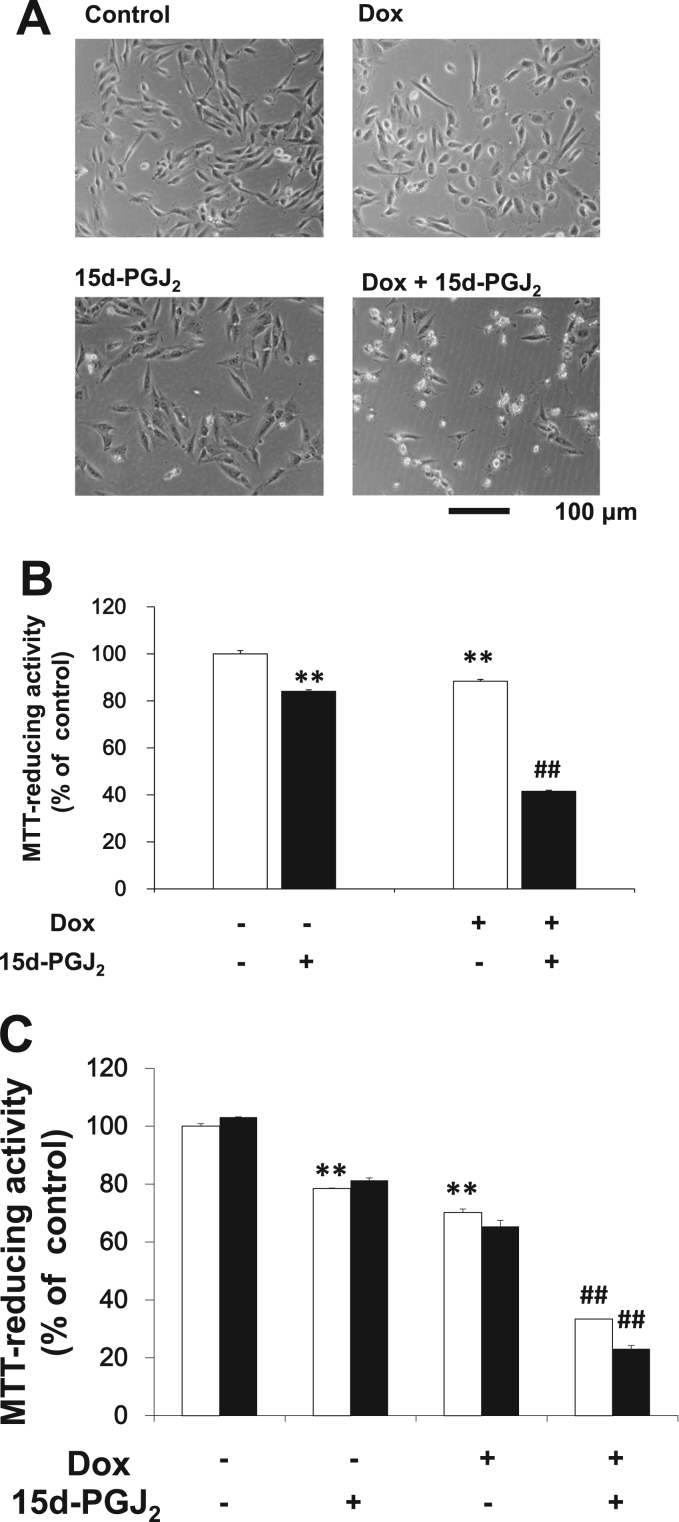
15d-PGJ_2_exacerbated the doxorubicin-disrupted morphology in Caki-2 cells. Caki-2 was treated with 1 μM doxorubicin (Dox) in the absence or presence of 20 μM 15d-PGJ_2_ for 24 h. (A) Morphologies were photographed by phase contrast. Scale bar=100 µm. (B) Cell viabilities were determined by MTT reducing activity. (C) Caki-2 cells were treated with 1 μM doxorubicin and/or 20 μM 15d-PGJ_2_ in the absence (open columns) or presence (filled columns) of 10 μM GW9662. Cell viabilities were determined by MTT reducing activity. Data are expressed as means±SE. (n =6). ^*^^*^P<0.01, compared with control. ##P<0.01, compared with 15d-PGJ_2_ alone.

As shown in Fig. 1, we evaluated cytotoxicities of doxorubicin and 15d-PGJ_2_ on Caki-2 cells with morphology and MTT-reducing activity (Fig. 1). In control culture, most Caki-2 cells were bipolar. Several cells were multipolar and have protrusions. Caki-2 cells have elongated shapes, and grow attached to a substrate (Fig. 1A). Doxorubicin exhibited weak toxicities at 1 μM alone (Fig. 1B). It decreased bipolar cells and increased polygonal cells (Fig. 1A). 15d-PGJ_2_ also reduced cell viability slightly at 20 μM alone (Fig. 1B). In contrast to doxorubicin, 15d-PGJ_2_ kept Caki-2 cells elongated shape, increased protrusions and made focal contacts clear (Fig. 1A). Combination of doxorubicin with 15d-PGJ_2_ caused cell death markedly (Fig. 1B). In the two anti-cancer agents-treated culture, Caki-2 cells were spherical in shape and peeled in suspension without attaching to a substrate. Filamentous protrusions were extended from residual cells (Fig. 1A).

PPARγ do not mediate the anti-tumor activity of 15d-PGJ_2_ in Caki-2 cells [11], [12], [17]. We confirmed that PPARγ antagonist, GW9662, did not rescued Caki-2 cells from the cytotoxicity of 15d-PGJ_2_ (Fig. 1C). Similarly, GW9662 did not affected viability of Caki-2 cells in doxorubicin-treated Caki-2 cells regardless 15d-PGJ_2_.

### 3.2 15d-PGJ_2_ and doxorubicin induced apoptosis synergistically in Caki-2 cells

**Fig. 2:**
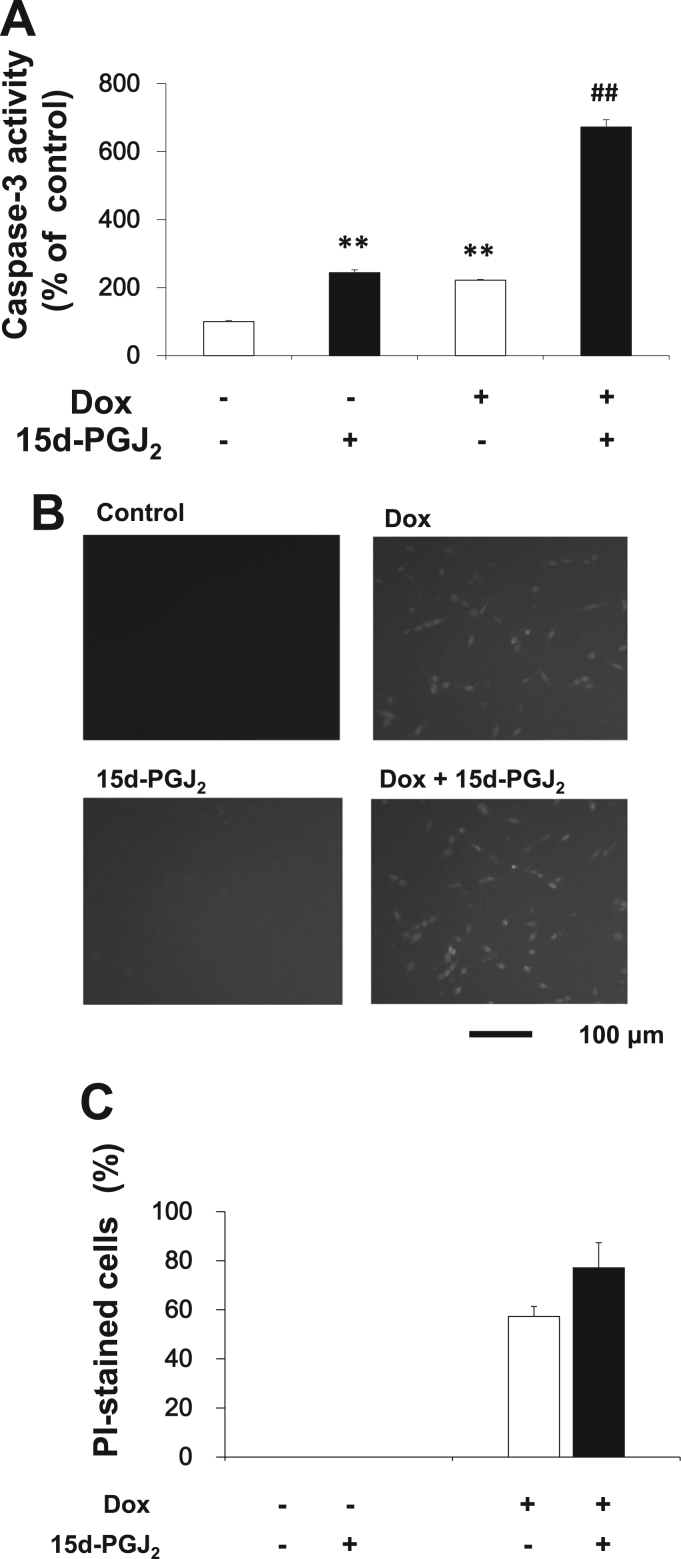
15d-PGJ_2_ and doxorubicin induced apoptosis synergistically in Caki-2 cells. Capase-3: Caki-2 cells were treated with 1 μM doxorubicin in the absence or presence of 20 μM 15d-PGJ_2_. Caspase-3 activities were determined as initial velocities (A). Data are expressed as means±SE. (n=3). ^*^P<0.05, compared with control. ##P < 0.01, compared with 15d-PGJ_2_ alone. PI staining: Caki-2 was treated with 5 μM doxorubicin in the absence or presence of 20 μM 15d-PGJ_2_ for 24 h. PI-stained Caki-2 cells were photographed (B) and counted (C). Scale bar=100 µm. Cell death is expressed as % (PI-stained cell numbers/total cell numbers). Data are expressed as means±SE. (n=12). Since PI-stained cells were not detected in the control culture, significant difference compared with the control culture could not be tested.

15d-PGJ_2_-induced apoptosis is accompanied with the caspase activation in Caki-2 cells. We confirmed that 20 μM 15d-PGJ_2_ activated caspase-3 significantly. On the other hand, 1 μM doxorubicin did not activate caspase-3. However, combination of doxorubicin with 15d-PGJ_2_ enhanced caspase-3 activity markedly (Fig. 2A).

Apoptosis is also characterized by PI staining of nuclei. PI is membrane impermeable and generally excluded from viable cells. When plasma membrane is damaged, PI is incorporated into cells and binds to DNA by intercalating between the bases in the nuclei. PI is commonly used for identifying dead cells in a population and as a counterstain in multicolor fluorescent techniques. We have not succeeded in detecting PI-stained nuclei of RCC treated with 1 μM doxorubicin and/or 20 μM 15d-PGJ_2_ (data not shown). Therefore, we performed PI staining of RCC treated with 5 μM doxorubicin and/or 20 μM 15d-PGJ_2_ (Fig. 2B and C). Little PI-positive cell was detected in control culture (Fig. 2B and C). PI-positive cells were increased in the doxorubicin-treated culture, but not in the 15d-PGJ_2_-treated one. Doxorubicin enhanced the PI-staining of 15d-PGJ_2_-treated cells slightly, but not significant.

### 3.3 A PI3K inhibitor increased the cytotoxicity of doxorubicin in Caki-2 cells additively

**Fig. 3:**
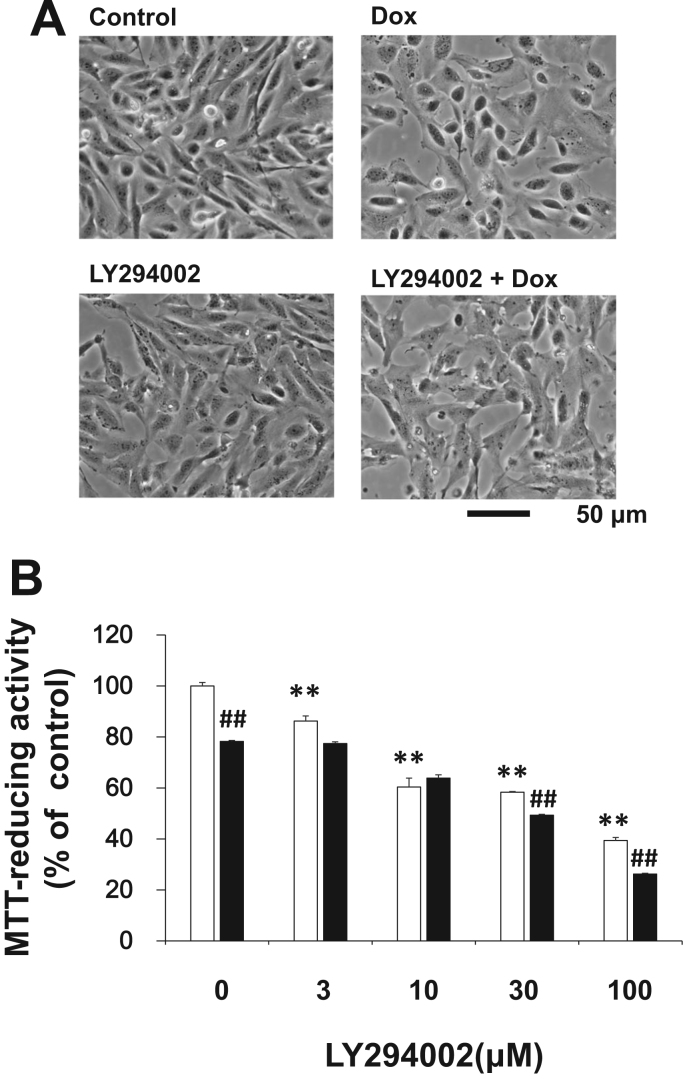
Additive effect of a PI3-K inhibitor on the anti-tumor activity of doxorubicin in Caki-2 cells. Caki-2 cells were treated with LY294002 at the indicated concentrations in the absence (open columns) or presence (filled columns) of 1 μM doxorubicin for 24 h. (A) Morphologies of RCCs treated with 30 μM LY294002 and/or 1 μM doxorubicin were photographed by phase contrast. 30 μM LY294002 1 μM doxorubicin Scale bar=50 µm. (B) Cell viabilities were determined by MTT reducing activity. Data are expressed as means±SE. (n=6). ^*^^*^P<0.01, compared with control. ##P<0.01, compared with LY294002 alone.

**Fig. 4:**
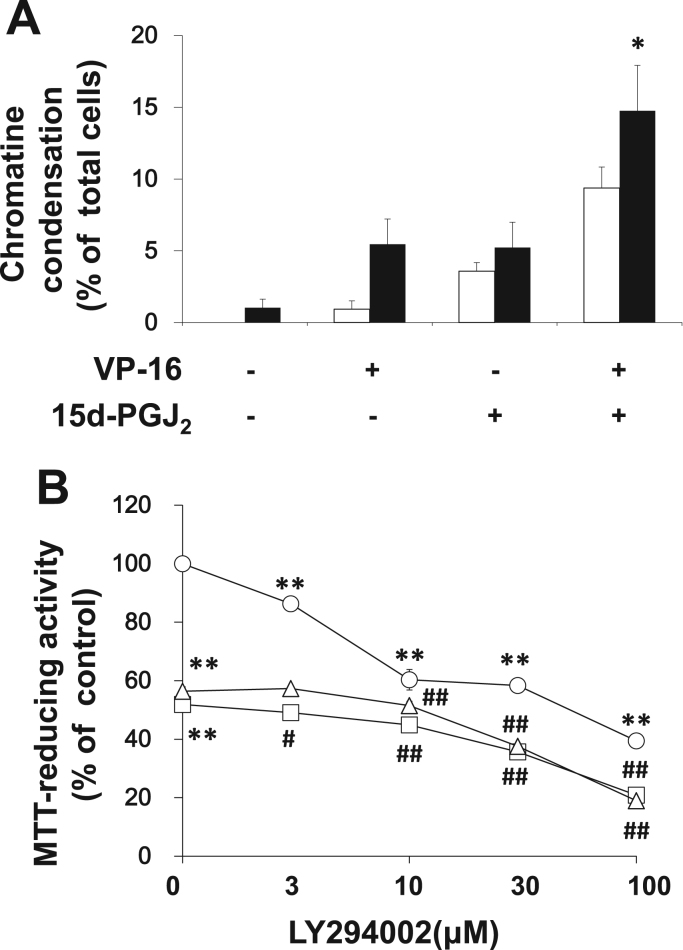
Additive effect of and 15d-PGJ_2_ and PI3-K inhibitor on the anti-tumor activity of topoisomerase inhibitors in Caki-2 cells. (A) Caki-2 cells were assayed for nuclear chromatin condensation following treatment for 20 h (open columns) or 24 h (filled columns) with 20 μM 15d-PGJ_2_ in the absence or presence of 70 μM VP-16. Data are expressed as means±SE. (n=12). Since chromatin-condensed nucleus were not detected in the control culture at 20 h, significant difference compared with the control culture could not be tested. ^*^P<0.05, compared with control (24 h). (B) Caki-2 cells were treated with LY294002 at the indicated concentrations in the absence (circles) or presence of 1 μM camptothecin (triangles) or 30 μM VP-16 (squares) for 24 h. Cell viabilities were determined by MTT reducing activity. Data are expressed as means±SE. (n=6). ^*^^*^P<0.01, compared with control. ##P<0.01, compared with camptothecin or VP-16 alone.

Previously, we have reported that Akt was involved in the cytotoxicity of 15d-PGJ_2_ [17]. As shown in Fig. 4A, we ascertained whether PI3K was involved in the synergy between 15d-PGJ_2_ and doxorubicin. The cytotoxicity of doxorubicin and a PI3K inhibitor, LY294002, on Caki-2 cells was evaluated with morphology and MTT-reducing activity. In control culture, most Caki-2 cells were bipolar. Several cells were multipolar and have protrusions. Caki-2 cells have elongated shapes, and grow attached to a substrate (Fig. 3A). The PI3K inhibitor, LY294002, induced cell death in a concentration-dependent manner (Fig. 3B). The PI3K inhibitor, LY294002, reduced the density of Caki-2 cells. In contrast to 15d-PGJ_2_, LY294002 did not degenerate morphology of Caki-2 cells significantly. In the presence of LY294002, doxorubicin reduced the density of Caki-2 cells and degenerated cell morphology markedly. However, effects of LY294002 on the cytotoxicity of doxorubicin was additive at the indicated concentrations, but not synergistic (Fig. 3A and B).

### 3.4 Doxorubicin did not condense chromatin in Caki-2 cells

Apoptosis is accompanied with chromatin condensation. As shown in Fig. 4A, 15d-PGJ_2_ condensed chromatin in RCCs [17] and neurons [22]. Doxorubicin acts the anti-cancer agent via inhibiting topoisomerase II. Previously, we have reported that a topoisomerase I inhibitor, camptothecin, condensed chromatin as well as 15d-PGJ_2_ [11]. A topoisomerase II inhibitor, etoposide (VP-16), -induced cell death [12] was also accompanied with chromatin condensation in Caki-2 cells (Fig. 4A). Furthermore, 20 μM 15d-PGJ_2_ increased the etoposide-condensed chromatin markedly. However, doxorubicin did not increase chromatin condensation regardless of 20 μM 15d-PGJ_2_ under the cytotoxic condition (1 μM, 24 h).

### 3.5 A PI3K inhibitor increased the cytotoxicity of doxorubicin in Caki-2 cells additively

Previously, we have reported the synergistic effects of 15d-PGJ_2_ on anti-tumor activities of camptothecin [11] and etoposide [12]. Therefore, we ascertained whether PI3K was involved the synergy between 15d-PGJ_2_ and the two topoisomerase inhibitors (Fig. 4B). At the concentration less than 10 μM, LY294002 did not appear to affect the cytotoxicity of camptothecin and etoposide. At the concentration more than 30 μM, the PI3K inhibitor enhanced cytotoxicities of the two topoisomerase inhibitors additively, but not synergistically.

## 4 Discussion

In the present study, we provided the first evidence that 15d-PGJ_2_ enhanced anti-tumor activities of the anthracycline antibiotic, doxorubicin, in human renal cell carcinoma, Caki-2 cells. Doxorubicin degenerated morphologies of Caki-2 cells in a different fashion from 15d-PGJ_2_. 15d-PGJ_2_ targets the cytoskeleton protein, actin, resulting in alteration of cell morphologies [23]. Actin is one of adapter proteins, which mediates the intracellular domain of integrin binds to the cytoskeleton. Since this integrin-adapter protein (actin) -cytoskeleton complex forms the basis of a focal adhesion, it was likely that 15d-PGJ_2_ increased protrusions and made focal adhesion clear. In contrast to 15d-PGJ_2_, doxorubicin appeared to enlarge the area of cytosol in comparison with that of nucleus. Doxorubicin altered morphologies of Caki-2 cells similarly to etoposide (VP-16) in accordance with the fact that they are topoisomerase II inhibitors [12].

15d-PGJ_2_-induced apoptosis was accompanied with chromatin condensation and caspase- 3 activation, and restored by co-treatment with a pan-caspase inhibitor, Z-VAD-FMK [17]. In the present study, we confirmed that 15d-PGJ_2_ activated caspase-3 and induced apoptosis in Caki-2 cells. In addition, 15d-PGJ_2_ enhanced the doxorubicin-activated caspase- 3 synergistically. Doxorubicin has also been reported to condense chromatin in myeloma and leukemia [24]. However, in Caki-2 cells, VP16 caused chromatin condensation, but doxorubicin did not under the condition exhibiting cytotoxicity. Topoisomerase II is the common target for doxorubicin and VP16. However, doxorubicin is the anthracycline antibiotic, whereas VP16 is the alkaloid derived from plants. Because of different basic structures, the two inhibitors did not always exhibit cytotoxicity in the same fashion in Caki-2 cells. To cite another case, doxorubicin elevated the permeability of plasma membrane, but etoposide did not under the condition exhibiting cytotoxicity (data not shown).

In comparison with normal tissues, 15d-PGJ_2_ are markedly decreased in tumors and metastatic breast tissues, suggesting that the reduction of 15d-PGJ_2_ affects the development of cancer and its progression to metastasis [25]. 15d-PGJ_2_ possesses opposite functions, cytoprotective activities and proapoptotic properties in many cancer cell lines [26]. 15d-PGJ_2_ induces apoptosis PPARγ-dependently in neuroblastoma [27] and hepatic myofibroblasts [28], whereas PPARγ-independently in colorectal cancer [29], breast cancer [30], hepatocellular carcinoma [31] and prostate and bladder carcinoma [32]. We confirmed that PPARγ did not mediate the anti-tumor activity of 15d-PGJ_2_ in RCCs [11], [12], [16], [17]. In addition, PPARγ antagonist, GW9662, did not suppressed the combined cytotoxicity of 15d-PGJ_2_ and doxorubicin. These findings suggested that PPARγ did not mediate synergistic effects of 15d-PGJ_2_ and doxorubicin.

Previously, we have reported that the PI3K-Akt signaling played an important role in the cytoprotection and proliferation of RCCs [17]. 15d-PGJ_2_ markedly decreased the phosphorylation of Akt. The Akt inhibitor showed cytotoxicity with a low IC_50_ value, suggesting that 15d-PGJ_2_ exerted cytotoxicity via the inactivation of Akt. In the present study, we confirmed that the PI3K inhibitor mimicked the anti-tumor activity of 15d-PGJ_2_. However, we could not detect the synergistic effect between doxorubicin and PI3K inhibitor. In addition, the PI3K inhibitor did not enhanced cytotoxicities of another topoisomerase II inhibitor, etoposide, and a topoisomerase inhibitor I, camptothecin. 15d-PGJ_2_ has been reported to inhibit the ubiquitin-proteasome pathway in neuroblastoma cells [33]. The proteasome inhibitor potentiates the growth inhibition by doxorubicin in leukemia [24]. In Caki-2 cells, we could not rule out the possibility that 15d-PGJ_2_ might potentiate the anti-proliferative effect of doxorubicin via suppression of the ubiquitin-proteasome pathway. Thus, 15d-PGJ_2_ increased the chemosensitivity of doxorubicin independently of PPARγ and PI3K. Further studies are required to identify targets for 15d-PGJ_2_, which reduces the chemoresistance of doxorubicin.
